# Protective strategies against occupational stress among health professionals during the COVID-19 pandemic

**DOI:** 10.47626/1679-4435-2022-1016

**Published:** 2024-09-24

**Authors:** Paula de Souza Marinho, Luciana Valadão Vasconcelos Alves, Tiago Gomes Barroso Carvalho, Magda Guimarães de Araújo Faria

**Affiliations:** 1 Occupational Health, Instituto Federal de Educação, Ciência e Tecnologia Fluminense, Macaé, Rio de Janeiro (RJ), Brazil; 2 School of Nursing, Universidade do Estado do Rio de Janeiro (UERJ), Rio de Janeiro (RJ), Brazil

**Keywords:** occupational stress, occupational health, health personnel, COVID-19, estresse ocupacional, saúde do trabalhador, pessoal de saúde, COVID-19

## Abstract

The purpose of this study was to investigate which protective strategies against occupational stress were developed for health professionals during the COVID-19 pandemic. This was an integrative literature review conducted in 2021. The Medical Literature Analysis and Retrieval System Online and the Latin American and Caribbean Literature in Health Sciences databases were searched using the Boolean operator AND and the controlled vocabularies “health professionals” AND “occupational stress” AND “COVID-19”, both in English and Portuguese. After applying the inclusion criteria and reading the selected articles, a final sample of 24 articles was obtained. Protective strategies against occupational stress developed by institutions included psychological support, support from managers and team leaders, provision of personal protective equipment, appropriate schedules and workload, and training. As for the strategies developed by health professionals themselves, these included resilience, peer support, and self-care. Several protective strategies during the pandemic were observed, but institutions still need to develop and/or improve practices to offer better psychological conditions to health professionals in general.

## Abstract (pt)

O objetivo deste estudo é investigar as estratégias de proteção ao estresse ocupacional em profissionais de saúde na pandemia de covid-19. Trata-se de uma revisão integrativa da literatura realizada nas bases de dados Medical Literature Analysis and Retrieval System Online e Literatura Latino-Americana e do Caribe em Ciências da Saúde em 2021. Utilizou-se o operador booleano AND e vocabulários controlados, a citar: profissionais de saúde AND estresse ocupacional AND COVID-19 e suas respectivas equivalências com termos em inglês. Após a aplicação dos critérios de inclusão e leitura do material, obteve-se uma amostra final de 24 artigos. Suporte psicológico, apoio de gestores e líderes das equipes, disponibilidade de equipamentos de proteção individuais, cronogramas e carga horária apropriados e treinamentos foram estratégias de proteção ao estresse ocupacional organizadas pelas instituições. Quanto à resiliência, o apoio entre pares e os cuidados pessoais foram estratégias criadas pelos profissionais de saúde. Evidenciou-se o uso de diversas estratégias no cenário pandêmico, porém ainda se torna necessário que as instituições elaborem e/ou aprimorem suas práticas de modo a oferecer melhores condições psicológicas aos profissionais de saúde.

## INTRODUCTION

The severe acute respiratory syndrome coronavirus 2 (SARS-CoV-2) is the etiologic agent of the disease that caused the largest biopsychosocial emergency in the 21st century: COVID-19.^1^ It has claimed the lives of 6 million people worldwide.^2^

The coronaviruses were discovered in the 1960s. They are a class of several RNA viruses found in a wide range of species, including birds, camels, cats, bats, and humans. Coronaviruses can cause respiratory, hepatic, gastrointestinal and neurological diseases.^3,4^ They belong to the *Coronavidae* family, which has two subfamilies: *Orthocoronavirinae* and *Torovirinae.* The subfamily *Orthocoronavirinae* has four genera: alphacoronavirus, betacoronavirus, gammacoronavirus and deltacoronavirus. SARS-CoV-2 belongs to the betacoronavirus genre.^4^

A series of cases of patients presenting with acute viral pneumonia was observed in Wuhan, China, prompting genetic sequencing of the virus, which detected a new coronavirus, namely SARS-CoV-2.^3^ SARS-CoV-2 spreads rapidly through human contact, and it continues to cause infections more than 2 years after its discovery due to difficulties in controlling transmission, despite the availability of different vaccines. Additionally, the emergence of more transmissible and antibody-resistant variants has increased the duration of the pandemic. Researchers believe that some properties of the coronavirus, such as high dissemination, genetic diversity, ability to recombine genomes and biological interactions between humans and animals, may lead to periodic infections caused by new coronaviruses.^1^

Health professionals have suffered many physical, psychological, and social consequences due to the pandemic, as they are directly confronted with the challenges posed by this scenario on a daily basis. In Wuhan, China, mental health issues among frontline health professionals during the pandemic were observed, impacting factors such as attention, comprehension, and decision-making abilities, thereby causing stress. This could hinder the fight against SARS-CoV-2 and have prolonged effects on the health of these professionals.^5^

To face adversities and risks in the workplace, institutions and professionals have developed protective strategies, consisting of actions aimed at minimizing the occurrence of occupational accidents or diseases. As such, these protective strategies, ranging from specialized support to the use of personal protective equipment (PPE), allow professionals to stay safe and comfortable while working.

Work can be a source of either happiness or suffering, and, in the face of unfavorable circumstances, workers can develop protective strategies to defend against and avoid the onset of suffering and illness.^6^

Protective strategies, such as social and family support, spirituality, and resilience, decrease the likelihood of developing mental illness by up to 6 times.^7^ This underscores the relevance of protective factors developed and implemented by workers, especially health professionals working during the pandemic.

The importance of this study lies in the need of updated reviews on this topic, as they are important for atypical situations. Due to the historic context characterized by a high global production and transmission of information, synthesizing knowledge into one study allows its systematization, which facilitates the access and contributes to awareness not only among health professionals, but also population in general.

Additionally, this study supports the development and implementation of protective initiatives against occupational stress, as mental health care is important for the well-being of health professionals and the control of the pandemic. Finally, it provides insight into gaps in the literature concerning important aspects of this theme and priorities for future studies.

Therefore, the aim of this study was to investigate protective strategies against occupational stress developed by health professionals and health care institutions during the COVID-19 pandemic.

## METHODS

This was an integrative review of the literature aiming at synthesizing evidence from scientific publications with different methodologies (qualitative and quantitative). The objective was to provide a theoretical and empirical basis for applying these studies in practice.^8^

Six steps for integrative reviews of literature were followed: 1. Formulating the guiding question; 2. Establishing inclusion, exclusion, and search strategy criteria; 3. Defining which information would be extracted and analyzed from the selected studies; 4. Evaluating manuscripts; 5. Interpreting results; 6. Presenting the synthesis of the results.^9^

The first step consisted of formulating the guiding question according to the PICo strategy (Population, Phenomenon of Interest and Context).^10^ Population (P) was defined as health professionals, the Phenomenon of Interest (I) was defined as protective strategies against occupational stress, and the Context (Co) was defined as the COVID-19 pandemic. Thus, we formulated the following question: Which protective strategies against occupational stress have been adopted or provided for health professionals during the COVID-19 pandemic?

The second step involved the definition of the inclusion criteria as follows: full-text research articles freely available online, published in Portuguese, English, and/or Spanish from 2020 to 2021. Literature reviews, commentary/reflection articles, editorials, studies whose data were collected prior to the COVID-19 pandemic, studies that were not pertinent to the topic, and duplicates were excluded.

The Biblioteca Virtual em Saúde (BVS) via Medical Literature Analysis and Retrieval System Online (MEDLINE) and the Latin American and Caribbean Health Sciences Literature (LILACS) databases were searched using the Boolean operator “AND” and the controlled vocabularies “Health professionals”, “Occupational health”, and “COVID-19”, both in Portuguese and English. Thus, the following search strategy was used: (Profissionais de Saúde) AND (Estresse Ocupacional) AND (COVID-19); and (Health Professionals) AND (Occupational Stress) AND (COVID-19).

The third step consisted of categorizing the data extracted from the selected articles.^9^ A worksheet was organized using Microsoft Excel 2016, including information such as title, country and year of publication, protective strategies against occupational stress presented in the study, and journal of publication.

**Figure 1:**
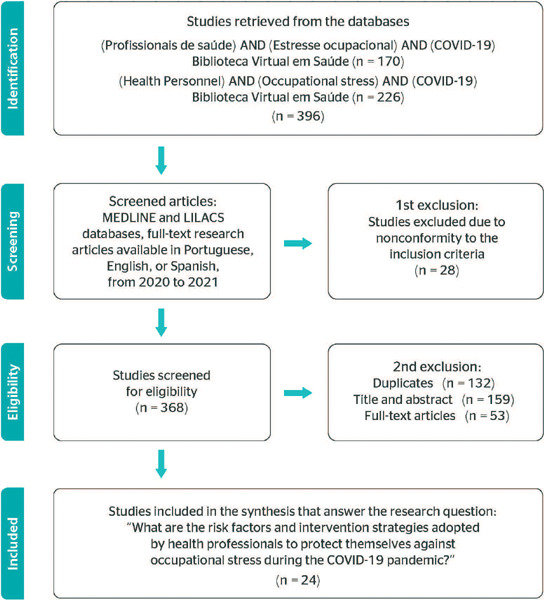
Flowchart of the selection process - Brazil, 2021. LILACS = Latin American and Caribbean Health Sciences Literature; MEDLINE = Medical Literature Analysis and Retrieval System Online.

In step four, the principles of the Preferred Reporting Items for Systematic reviews and Meta-Analyses (PRISMA) were used for the analysis of selected articles, involving identification – total of studies found in each database; screening – total of studies selected and excluded through filters of the digital library; eligibility – total of studies selected and excluded after full-text analysis; and inclusion – total of studies included in the qualitative synthesis (Figure 1).^11^

After inserting the controlled vocabularies in the databases, the inclusion criteria were applied using the digital library filters, allowing the selection of full-text articles available online in Portuguese, English, or Spanish, published from 2020 to 2021. After duplicate removal, the titles and abstracts of selected articles were read, and the inclusion and exclusion criteria were reapplied. The remaining articles were read in full and selected according to their relevance to the topic of research.

To organize the filtering process, the articles were inserted into the worksheet developed during the third step. Additionally, the reference manager Mendeley (version 1.19.4) was used to facilitate the organization, analysis, and selection of studies.

In the fifth step, the studies were analyzed in a descriptive and interpretative manner, considering ethical aspects and respecting ideas, concepts, and definitions of authorship. Finally, the sixth and last step consisted of writing the manuscript, including all research steps and the synthesis of the results found.

## RESULTS

The initial database search retrieved a total of 396 articles. During the first exclusion step, automatic filters available on the BVS website were used with the inclusion criteria. Twenty-eight publications were excluded, leaving 368 articles for the second step. In this step, 132 duplicates were excluded and, after reading titles and abstracts, other 159 publications were excluded according to the exclusion criteria. The remaining 53 articles were read in full, of which only 24 were considered adequate for inclusion in this study.^12,13,14,15,16,17,18,19,20,21,22,23,24,25,26,27,28,29,30,31,32,35^ Details of the article selection process are shown in Figure 1, and the synthesis of the research results is presented in Chart 1.

**Chart 1:** Synthesis of included studies, Brazil, 2021

Title	Year/country	Journal	Strategies
A large scale of nurses participated in beating down COVID-19 in China: the physical and psychological distress^12^	China, 2021	Current Medical Science	Improvement of work organization and work conditions; peer support.
An innovative wellness partner program to support the health and well-being of nurses during the COVID-19 pandemic: implementation and outcomes^13^	United States, 2020	Nursing Administration Quarterly	Psychological support program.
Battle buddies: rapid deployment of a psychological resilience intervention for health care workers during the COVID-19 pandemic^14^	United States, 2020	Anesthesia & Analgesia	Psychological support program; resilience; provision of PPE.
Benefits of auriculoacupuncture in nursing professionals working at COVID-19 in light of the Comfort Theory^15^	Brazil, 2020	Escola Anna Nery	Auriculoacupuncture.
COVID-19 pandemic: looking after the mental health of our healthcare workers^16^	United Kingdom, 2020	Journal of Occupational and Environmental Medicine	Psychological support; peer support.
Depression and anxiety in healthcare professionals during the COVID-19 pandemic^17^	Germany, 2021	Epidemiology and Infection	Psychological support.
Ethical and psychosocial considerations for hospital personnel in the Covid-19 crisis: moral injury and resilience^18^	Austria, 2021	PLoS ONE	Resilience; peer support; institutional leadership; psychological support; provision of PPE.
Experience of 2003 SARS has a negative psychological impact on healthcare workers in the COVID-19 pandemic: a cross-sectional study^19^	China, 2021	Sao Paulo Medical Journal	Online mental health service.
Investigation of mental health among hospital workers in the COVID-19 pandemic: a cross-sectional study^20^	Turkey, 2020	Sao Paulo Medical Journal	Psychological support.
Mental health providers during COVID-19: essential to the US Public Health workforce and in need of support^21^	United States, 2021	Public Health Reports	Psychological support.
Mental health status among Chinese healthcare-associated infection control professionals during the outbreak of coronavirus^22^	China, 2021	Medicine	Improvement of work organization and work conditions; breathing techniques.
Mental health status of health-care professionals working in quarantine and non-quarantine Egyptian hospitals during the COVID-19 pandemic^23^	Egypt, 2020	Eastern Mediterranean Health Jornal	Psychological support; peer support; religious practices; adequate sleep and rest.
Nurse reports of stressful situations during the COVID-19 pandemic: Qualitative analysis of survey responses^24^	United States, 2020	International Journal of Environmental Research and Public Health	Peer support; psychological support; provision of PPE.
Occupational stress and mental health among anesthetists during the COVID-19 pandemic^25^	Italy, 2020	International Journal of Environmental Research and Public Health	Improvement of work organization and work conditions; healthy eating habits.
Palestinian health care workers’ stress and stressors during COVID-19 pandemic: a cross-sectional study^26^	Palestine, 2020	Journal of Primary Care & Community Health	Psychological support; peer support; religious practices; provision of PPE.
Preventing and addressing the stress reactions of health care workers caring for patients with COVID-19: development of a digital platform (Be + Against COVID)^27^	Spain, 2020	JMIR mHealth and uHealth	Online mental health service.
Psychological distress among health service providers during COVID-19 pandemic in Nepal^28^	Nepal, 2021	PLoS ONE	Provision of PPE.
Psychological impact of the COVID-19 pandemic on healthcare workers at acute hospital settings in the SouthEast of Ireland: an observational cohort multicentre study^29^	Ireland, 2020	BMJ Open	Psychological support; online mental health service; improvement of work organization and work conditions.
Psychological stress among health care professionals during the 2019 novel coronavirus disease outbreak: cases from online consulting customers^30^	China, 2020	Intensive & Critical Care Nursing	Online mental health service.
Psychological stress risk factors, concerns and mental health support among health care workers in Vietnam during the coronavirus disease 2019 (COVID-19) outbreak^31^	Vietnam, 2021	Front Public Health	Psychological support; online mental health services.
Psychosocial burden of healthcare professionals in times of COVID-19 - a survey conducted at the University Hospital Augsburg^32^	Germany, 2020	German Medical Science	Psychological support; resilience; peer support; improvement of work organization and work conditions.
The combined effect of perceived COVID-19 infection risk at work and identification with work community with psychosocial wellbeing among Finnish social sector and health care workers^33^	Finland, 2020	International Journal of Environmental Research and Public Health	Peer support; provision of PPE.
Work-related and personal factors associated with mental well-being during the COVID-19 response: Survey of health care and other workers^34^	United States, 2020	Journal of Medical Internet Research	Resilience; organizational leadership; peer support.
Work-related challenges among primary health centers workers during COVID-19 in Saudi Arabia^35^	Saudi Arabia, 2021	International Journal of Environmental Research and Public Health	Psychological support; peer support.

PPE = personal protection equipment.

## DISCUSSION

### PROTECTIVE STRATEGIES AGAINST OCCUPATIONAL STRESS: PSYCHOSOCIAL SUPPORT

The recommendation of strategies related to psychosocial support as a protective measure against occupational stress among health professionals during the COVID-19 pandemic was identified in several studies.

The implementation of psychological support programs stands out.^13,14,27^ In one study, nurses and graduate nursing students developed a program aiming at providing advice on health and wellness actions that prioritized physical exercise, healthy eating habits, sleep, and stress control. The participants were nurses working in the frontline against COVID-19 in the United States, and they reported benefits in physical and mental health through the promotion of self-care.^13^ Psychological support programs offered by employers or philanthropic institutions are important for providing active listening and guidance, in addition to facilitating professionals’ access to specialized support, as many are overwhelmed and demotivated and lack financial resources to seek this type of support.

Studies have shown that health professionals working during the pandemic should seek qualified professional support to decrease occupational stress^17,19,21,23,26,29,31,35^ and have also highlighted the importance of employers offering psychological support.^16,18,24,31^

Conversely, one of the studies conducted in Germany identified that, after the pandemic started, most health care professionals did not seek professional support because they believed there was limited psychological support available, and they were not the ones who needed it the most. Also, they reported difficulty in access because they were not suffering enough to seek help. Thus, researchers suggest that psychological support should be focused on the needs of each team and flexible to accommodate the staff’s schedule.^17^

Resilience^18,32,34^ and peer support^12,16,18,23,24,26,33,34,35^ were also mentioned as protective strategies against occupational stress. A publication describes a psychological resilience intervention based on a peer support model and on the incorporation of “stress inoculation” methods.^14^ In a German study, peer psychological support was considered the most relevant strategy for reducing stress, exhaustion, and depression in 63% of the reports.^32^

The support and efforts made by institutional leadership from mental health professionals and peers result in improvements in health care staff cohesion and allow social exchange,^18,34^ providing relief from occupational stress. Furthermore, whenever possible, working in pairs promotes greater safety in handling PPE. Moreover, the possibility of shifting professionals and the support in decision-making are factors that reduce stress. Peer psychological support was also provided through proactive contact with those who remained in quarantine due to COVID-19 infection and sharing the experiences lived since then. For this, the maintenance of necessary sanitary measures was required in the pandemic context.^18^

According to a study,^18^ improvement in communication between managers and other employees results in better psychological conditions. Clear instructions on job procedures are responsible for more stable routines and work teams. Furthermore, the authors identified that hospitals with flexible structures and decentralized decision-making processes achieve better results than highly centralized organizations with management that does not understand the needs of their employees. The effective establishment of dialogue between managers and other health professionals reduces uncertainties, anxiety, and stress about the actions taken.^18^ Similarly, another article highlighted the importance of support from health professionals’ supervisors, which was associated with adequate levels of well-being, satisfaction, and engagement, as well as a decline in intentions to quit the job.^34^

Individual strategies were identified for dealing with occupational stress, such as connecting with family and friends and leisure time.^32^ Additionally, workers have become more attentive to sleep health and relaxation and general well-being practices.^23^ Healthy eating habits,^25^ as well as mindfulness practices, breathing techniques, and positive affirmations,^22^ are associated with better psychological conditions. Two studies, one from Egypt and another from Palestine, identified that health professionals working during the COVID-19 pandemic resorted to religious practices in order to reduce stress and anxiety. Moreover, the majority of health professionals incorporate other strategies in pursuit of mental balance, including listening to music, reading, and engaging in physical exercise.^23,26^ A study conducted in Brazil with 33 frontline professionals, consisting mostly of women (92.3%), nursing technicians (61.5%), and intensive care unit staff (50.0%), reported that auriculoacupuncture therapy has been beneficial in mitigating stressors, providing better levels of comfort, relief, and tranquility.^15^

Another protective measure mentioned were online mental health services, such as websites or applications that provide information, training, and virtual contact with specialized teams.^19,27,29,30,31^ A phone helpline for psychological emergencies was made available in one of the experiences.^19^ One of the studies revealed that health professionals with moderate stress levels preferred to access psychological support through websites, while health professionals with severe stress levels opted for professional support.^31^

The development of a web-based platform with psychological resources can help health professionals working during the COVID-19 pandemic to access knowledge and practices for stress reduction, as well as serve as a training space. Similar experiences have proven successful, such as Telessaúde, a project that offers distance learning courses, teleconsultation, and telediagnosis in primary care.^36^

Finally, a Chinese study revealed that 38% of health professionals reported subjective benefit and desire for additional online mental health services.^29^ However, a more in-depth evaluation of the effectiveness and long-term use of these online tools in reducing occupational stress and other mental distress is necessary. It is worth noting that individual interventions to enhance resilience in health professionals will be more effective if accompanied by institutional measures within health care institutions, aiming to reduce their burden.^25^

### PROTECTIVE STRATEGIES AGAINST OCCUPATIONAL STRESS: MEASURES ADOPTED BY INSTITUTIONS

Exposure to irregular schedules and exhausting workloads were significant risk factors for physical discomfort, stress, and decreased self-esteem among Chinese nurses working in hospitals, mobile care units, and shelters.^12^ In line with this, publications highlight that institutional administrations should implement a more efficient work system, balancing the occupational health of employees and improving human resources aspects to adapt to the current scenario.^12,18,22,24,24,26,28,29,32^ Therefore, work schedules and hours that align with occupational health guidelines (allowing sufficient time for rest and leisure) are necessary.

The availability of alternative accommodations that provide food and other necessary resources for professionals to temporarily isolate and recover from work are also cited as a psychological relief measure. This measure can reduce the risk of contagion, which is a predominant factor in the occupational stress of the studied professionals.^18^

The U.S. Centers for Disease Control and Prevention (CDC) recommend that health professionals use N95 respirators or equivalents with higher capacity for filtering biological agents.^37^ However, a lack of PPEs in institutions has been observed,^14,24,26,28,33^ which acts a significant stressor, turning urgent the availability according to the demand of these devices.^18,24^

In this review, we also showed that correct training on the use of PPE is essential, because its absence exposes the worker to occupational stress due to insecurity regarding protection against the virus.^28^ These instructions should be based on scientific and constantly updated evidence.^26^

## CONCLUSIONS

Since the beginning of the pandemic, health institutions have exposed their workers to biological risks as a result of the imminent threat of SARS-CoV-2 and other microorganisms. Additionally, workers face ergonomic risks characterized by excessive workload, ambiguity, and conflicts regarding roles, fear of COVID-19 infection, and deprivation of sleep and leisure. Furthermore, institutions with highly centralized decisions tend to prioritize less the well-being of their workers. Such factors leave health professionals more susceptible to diseases such as occupational stress.

The problems associated with occupational stress must be solved, and, in order to do so, institutions have to review which protective strategies they have to offer to their employees. Psychological support by mental health professionals and support by managers and team leaders are important measures to decrease occupational stress levels, as well as adequate PPE availability and instructions on its use, appropriate schedules, and effective training for each role. Moreover, providing accommodation for professionals to temporarily isolate is also a valid measure.

Health professionals also reinforce and create protective strategies against occupational stress in pandemic times through resilience and peer support. Self-care, such as balanced sleep, time for leisure, healthy eating habits, and religious practices, are effective to improve the mental health of professionals.

Finally, although this study shows the effective use of many protective strategies in a pandemic setting, institutions still need to develop and/or improve their practices in order to provide better psychological conditions to health professionals in general.
